# Fabrication and Characterization of Nanocomposite Flexible Membranes of PVA and Fe_3_O_4_

**DOI:** 10.3390/molecules26010121

**Published:** 2020-12-29

**Authors:** Belal Salah, Ahmad I. Ayesh

**Affiliations:** 1 Department of Math., Stat. and Physics, Qatar University, Doha 2713, Qatar; belal141991@gmail.com; 2 Center for Sustainable Development, Qatar University, Doha 2713, Qatar

**Keywords:** nanocomposite, metal oxide nanoparticles, flexible membranes, Fe_3_O_4_, PVA

## Abstract

Composite polymer membranes of poly(vinyl alcohol) (PVA) and iron oxide (Fe_3_O_4_) nanoparticles were produced in this work. X-ray diffraction measurements demonstrated the formation of Fe_3_O_4_ nanoparticles of cubic structures. The nanoparticles were synthesized by a coprecipitation technique and added to PVA solutions with different concentrations. The solutions were then used to generate flexible membranes by a solution casting method. The size and shape of the nanoparticles were investigated using scanning electron microscopy (SEM). The average size of the nanoparticles was 20±9 nm. Raman spectroscopy and Fourier-transform infrared spectroscopy (FTIR) were utilized to investigate the structure of the membranes, as well as their vibration modes. Thermal gravimetric analysis (TGA) and differential scanning calorimetry (DSC) demonstrated the thermal stability of the membranes and the crystallinity degree. Electrical characteristics of the thin membranes were examined using impedance spectroscopy as a function of the nanoparticles’ concentrations and temperatures. The resistivity of the fabricated flexible membranes was possible to adjust by controlled doping with suitable concentrations of nanoparticles. The activation energy decreased with the nanoparticles’ concentrations due to the increase in charge carriers’ concentrations. Therefore, the fabricated membranes may be applied for practical applications that involve the recycling of nanoparticles for multiple application cycles.

## 1. Introduction

Poly(vinyl alcohol) (PVA) is known as a water-soluble and synthetic polymer that is produced from poly(vinyl acetate) through a hydrolysis process [1,2]. PVA exhibits a long flexibility chain and a great concentration of polar groups conferring to a molar polymer’s mass. The PVA polymer is biocompatible and has superior adhesion characteristics; thus, its hydrogels are used for many biomedical applications ref. [3,4]. It also has many applications in the field of production of biodegradable blends, since it is water-soluble, easy to form thin films/membranes, and practical to produce natural blends by mixing it with other natural polymers [1,3,5].

However, the electrical conductivity of PVA is low, since it is a material of proton conduction [6], which limits its device applications in the pure form. Therefore, precise control of its electrical conductivity is essential to facilitate its utilization for practical device applications, including bioimplantable devices, resistive switching devices, and magnetic-controlled drug delivery devices [7,8,9]. Plasticizers, such as glycerol (GL), are a novel class of materials with many outstanding properties, including thermal steadiness, nonflammability, nondispersal, and extraordinary ionic conductivity [10]. Many plasticizers also have high electrical conductivity; thus, they can be used as additives to PVA to enhance and control its electrical conductivity [3,11,12]. Moreover, the doping of PVA membranes with GL enables the control of their flexibility and increases their durability [13].

Nanoparticles are grains of materials with size dimensions in nanometers, and they hold novel physical and chemical features that are dissimilar to their bulk form [14]. Therefore, controlled blending of PVA with nanoparticles permits to take advantage of the novel characteristics of nanoparticles to produce custom-designed nanocomposite membranes for device applications [15]. The characteristics of PVA membranes may be designed to target specific applications, according a thorough choice of additive nanoparticles’ sizes, types, concentrations, and shapes [11,16]. Those flexible membranes take advantage of the superior characteristics of nanoparticles while maintaining their flexibility and other attractive features [17]. Furthermore, using magnetic nanoparticles such as iron oxide (Fe_3_O_4_) allows the recycling of nanoparticles by dissolving PVA in water and retrieving the nanoparticles by a magnet [18]. This supports the green utilization of nanoparticles where they can be employed for numerous application cycles [19]. The retrieved nanoparticles can be used for the generation of new PVA-based membranes and apply them for further flexible device applications [11,12,15,17]. Recently, researchers synthesized Fe_3_O_4_ nanoparticles by a surfactant-free sonochemical reaction and added them to polyvinyl alcohol (PVA) to form flexible membranes [20]. They found that the nanoparticles enhanced both the thermal stability as well as the flame-retardant characteristics of the PVA matrix. Furthermore, a different investigation was performed on ribbons of Fe_3_O_4_ nanoparticles and PVA polymers with various concentrations [21]. Atomic force microscopy analysis revealed that the encapsulation of PVA with Fe_3_O_4_ decreased the agglomeration, controlled the morphology of nanoparticles to be further spherical, resulted in the further dispersion of nanoparticles, and decreased surface roughness.

To show that the Fe_3_O_4_ nanoparticles can potentially aid as a carrier of the protein that keeps the antigenicity of a conjugate, a method to synthesize 3-aminepropyltrimethoxysilane-PVA-magnetite nanoparticles altered using antiprotein kinase C (PKC)α [22]. The conjugate includes Fe_3_O_4_ nanoparticles that are bound covalently to the antibody: antiprotein kinase C (PKC)α. The conjugate process can help for localization of cellular PKC, as well as the inhibition of its function. The action of anti-PKCα conjugated via Fe_3_O_4_ was verified by recognizing PKCα by the method of Western blot.

The utilization of flexible membranes for device applications requires detailed identification of their electrical characteristics, including phase and structure transition, electrical conductivity, and polarization mechanisms, as well as charge transport [23,24]. Here, impedance spectroscopy represents an exceptional tool that provides insight into polymer membrane characteristics [25]. It enables the identification of both the conductivity and capacitance of the produced membranes, the effect of grain boundaries, the impact of blocking electrodes, etc. [26].

The above investigations [20,21,22] did not examine the effects of the addition of Fe_3_O_4_ nanoparticles into PVA to enhance its electrical resistivity; neither had they tested the influence of Fe_3_O_4_ nanoparticles’ concentrations on adjusting the electrical resistivity of a PVA membrane. Therefore, this investigation presents the fabrication and thorough characterization of flexible composite membranes that include PVA, glycerol, and Fe_3_O_4_ nanoparticles. The influence of nanoparticles’ concentrations on the structure and electrical characteristics of the fabricated membranes are investigated. This work represents a continuation of our previous investigations of producing PVA nanoparticle composites for device applications ref. [11,12,15,17,27,28,29,30]. The previous investigations revealed that the modification of PVA with nanoparticles and plasticizers permit fine adjustments of the electrical and mechanical properties of its membranes.

## 2. Results and Discussion

**Figure 1:**
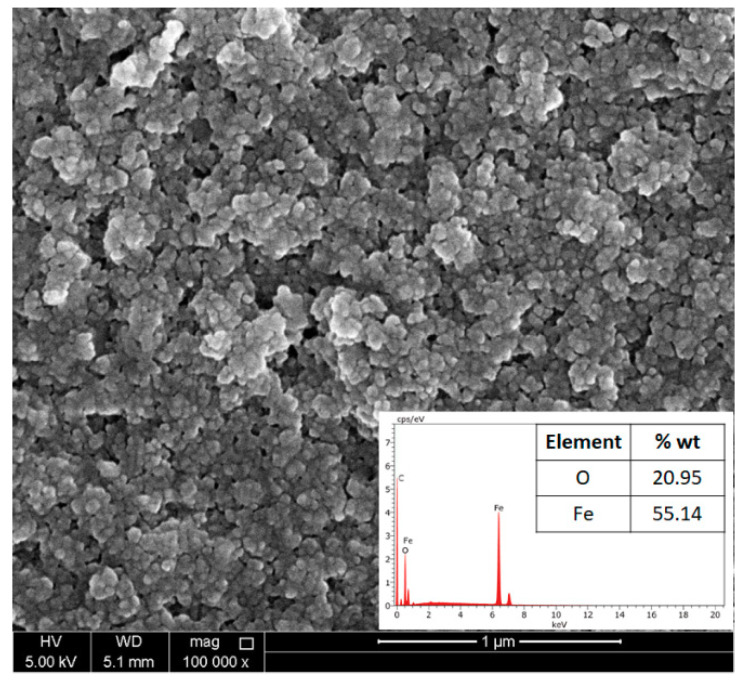
SEM image of the synthesized Fe_3_O_4_ nanoparticles. The inset is the energy-dispersive spectroscopy (EDS) analysis.

The morphology of the synthesized Fe_3_O_4_ nanoparticles was examined using SEM images, presented in Figure 1. The figure revealed an agglomerate of nanoparticles. The nanoparticle size was calculated from the SEM images, with an average size of 20±9 nm. The agglomeration of the nanoparticles was due to their magnetic nature (magnetic interaction). It should be noted that the SEM image was fuzzy due to imaging distortion, since the nanoparticles were magnetic. The composition of the generated nanoparticles was assured using the EDS measurements, as depicted in the inset of Figure 1.

**Figure 2:**
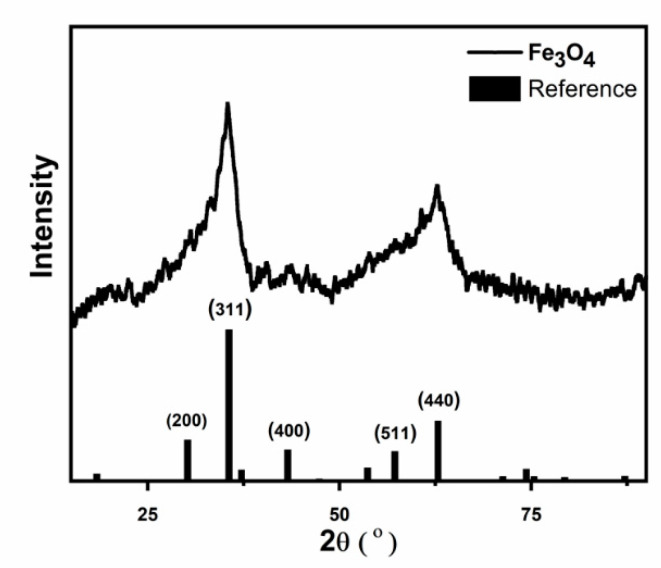
X-ray diffraction (XRD) analysis of Fe_3_O_4_ nanoparticles and the reference pattern according to ICSD:250540 and ICDD:98-025-0540 [31].

Figure 2 reveals the XRD analysis of the synthesized Fe_3_O_4_ nanoparticles. The figure emphasizes the formation Fe_3_O_4_ nanoparticles that exhibit cubic structures, as confirmed by the reference cards with numbers ICSD: 250540 and ICDD: 98-025-0540 [31]. Calculation of the degree of crystallinity was done by the equation [32]: $\%\;Crystallinity=\frac{total\;area\;of\;main\;peak\times 100}{Total\;area\;of\;all\;peaks}$%, and it yielded 1.5%. However, this value can be misleading, since many works have reported that the low intensity of the XRD spectrum of Fe_3_O_4_ nanoparticles is due to their magnetic nature [33]. The figure also reveals the reference pattern with Miller indices that are specified following the above structure. The XRD results agree well with the EDS measurements above.

**Figure 3:**
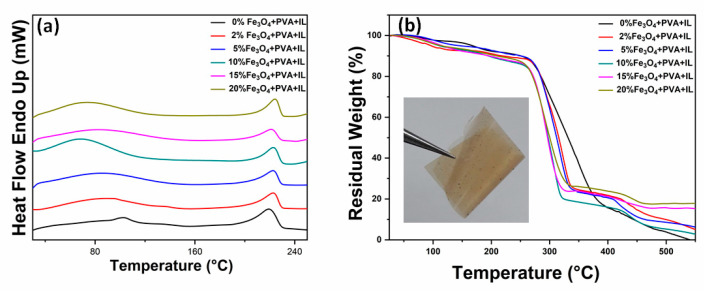
(**a**) Differential scanning calorimetry (DSC) thermograms and (**b**) thermal gravimetric analysis of the polyvinyl alcohol (PVA)-glycerol-Fe_3_O_4_ membranes at different wt% concentrations of nanoparticles. The inset in (**b**) shows a membrane with 10% Fe_3_O_4_. IL refers to glycerol in the legend.

The inset in Figure 3 reveals a picture of a PVA-GL-Fe_3_O_4_ (10%) membrane. The picture demonstrates its flexibility. The dark color is due to the nanoparticles. DSC measurements of the produced PVA-GL-Fe_3_O_4_ membranes are presented in Figure 3a, with a temperature up to 250 °C. During the heating of the membranes, two processes were observed [28]. The glass transition temperature (T_g_) shifted from 100 °C for pure PVA-glycerol to 70 °C with 20% of Fe_3_O_4_, indicating the relaxation of crystalline domains of PVA due to the nanoparticles’ inclusions inside the crystalline lattices. As the concentration of nanoparticles increased, the blocking of the crosslinking during the drying step of membranes increased, which led to a loosening molecular packaging. This effect, along with some large nanoparticles within the membranes not well-distributed, caused the shift of T_g_ to a low temperature [34]. The melting temperature (T_m_) slightly shifted from 220 to 225 °C due to the melting of the crystalline domains. However, the small shift means that the PVA polymer kept its crystallinity with the increasing nanoparticles’ concentrations. The shift was an indication of the crosslinking between the nanoparticles and PVA. This is also confirmed by both FTIR and Raman analyses below.

TGA analysis for the PVA-GL-Fe_3_O_4_ membranes was utilized to identify the stability and the loading amount of Fe_3_O_4,_ and its results are depicted in Figure 3b, with temperatures up to 550 °C. The figure revealed the moisture removal, degradation, and decomposition processes [28,30]. The lost weight until about 259 °C was due to the elimination of the moisture content. The degradation stages started from 259 °C to about 390 °C, and they were assigned to PVA chain degradations, while from 340 °C to 410 °C, they were assigned the carbonation. The final decomposition stage of the PVA chains started at around 430 °C. It should be noted that an error margin in wt% was due to losing nanoparticles during solution preparation and casting. The results revealed a small shift with increasing nanoparticle wt%, which meant a decent stability of the prepared membranes. In addition, the above findings were in agreement with other investigations that revealed that the addition of Fe_3_O_4_ nanoparticles to the PVA membranes enhances their thermal stability [20].

**Figure 4:**
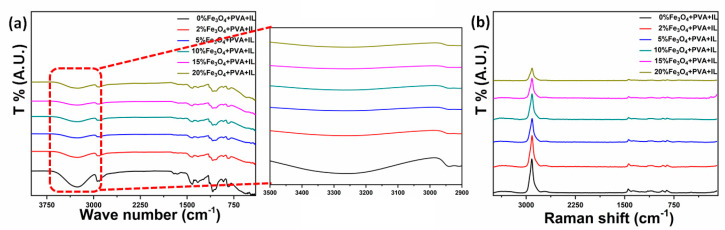
(**a**) Fourier-transform infrared (FTIR) and (**b**) Raman spectroscopy measurements of the PVA-glycerol-Fe_3_O_4_ membranes at different wt% concentrations of nanoparticles. IL refers to glycerol in the legend.

FTIR technique was used to examine the functional groups of PVA and the effects of Fe_3_O_4_ nanoparticles’ concentrations on them [29], as shown in Figure 4a. The broadband with a peak at 3268 cm^−1^ was consequent to the existence of the OH group of PVA. The 2907, 1100, and 1148–1709 cm^−1^ bands referred to C-H, C-O, and C-O-C stretching, respectively. Increased Fe_3_O_4_ nanoparticles’ percentages caused the intensity of the O-H and C-H peaks to decrease because of the inter or intra-molecular hydrogen bonding beside the complex formation of Fe_3_O_4_ nanoparticles with the OH groups of PVA [35]. However, it was observed that PVA kept its mechanical strength and elasticity. This conclusion was consist with other investigations that revealed that the addition of Fe_3_O_4_ nanoparticles did not influence the mechanical integrity of the PVA membranes [36].

Raman spectroscopy was utilized to estimate the crystallinity of the PVA-GL-Fe_3_O_4_ membranes. As shown in Figure 4b, the central peak was at 2914 cm^−1^, and it referred to the stretching vibration of CH_2_. The peaks at 1150 cm^−1^ and 1450 cm^−1^ were allocated to C-H and OH stretching vibrations in order [30]. The intensity of the peaks decreased with the nanoparticles’ concentrations and shifted to lower wavelengths, which indicated the decrease of the crystallinity degree of PVA due to its interactions with the nanoparticles.

**Figure 5:**
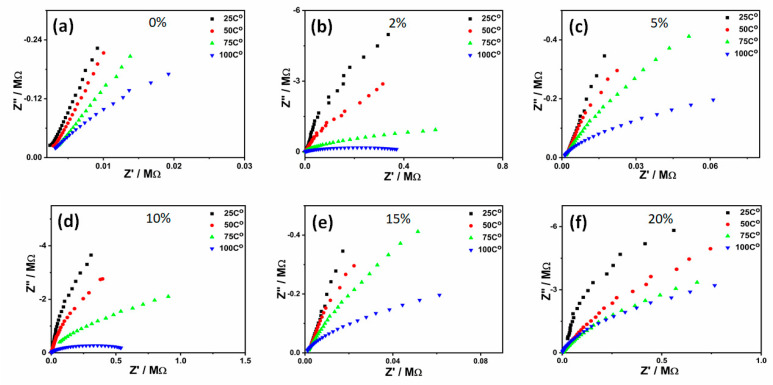
Electrical impedance measurements of the PVA-glycerol-Fe_3_O_4_ membranes at nanoparticles’ concentrations of (**a**) 0%, (b) 2%, (**c**) 5%, (**d**) 10%, (**e**) 15%, and (**f**) 20%.

Electrical impedance characterization as a function of the nanoparticles’ contents and temperatures were conducted for the PVA-GL-Fe_3_O_4_ membranes. Figure 5 illustrates Z’ versus Z” results for all prepared membranes at various nanoparticles’ concentrations and temperatures. The measurements presented in the figure can be extrapolated into semicircles, with the radius of each semicircle a demonstration of the membranes’ dc resistance. All figures revealed that increasing the temperature decreased the semicircle radius, referring to the decrease of the dc resistance and increase in the dc conductivity. This was mainly assigned to the increase of the carrier concentration, as well as the growing rate of electron transfer from valence to the conduction band [28]. At elevated temperatures, sufficient energy was attained by the ions, which empowered them to move and diffuse to the metal electrodes due to their potential differences [27]. The figure illustrates single semicircles for all measurements, which revealed that a membrane may be presented by a single parallel RC circuit [30]. The semicircle was assigned to the charge transfer via the kinetic process at high frequencies (at high frequencies, ions’ transfers were insignificant due to their relatively high relaxation time). Fitting the semicircles can be used to estimate the dc resistance and capacitance of each membrane. Here, the resistance and capacitance represented the effects of both the grain boundaries and depletion regions within each membrane [1,30].

**Figure 6:**
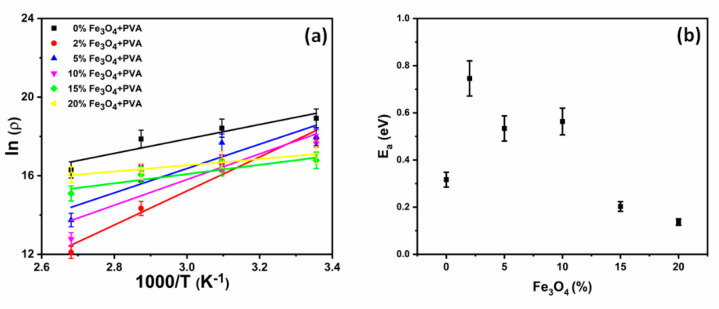
(**a**) The dependence of resistivity (natural logarithm) on inverse temperatures (1000/T) for the PVA-glycerol-Fe_3_O_4_ membranes at different wt% concentrations of nanoparticles, and (**b**) the dependence of the activation energy on the wt% concentrations of the nanoparticles.

The dc resistances (R) extracted from the impedance results in Figure 5 can be used to evaluate the electrical resistivity using the equation: $=\frac{Rl}{A}$, where l represents the membrane thickness and A is the cross-section area of the electrical electrode. Figure 6a reveals the dependence of the resistivity (natural logarithm) on the inverse temperature for the PVA-GL-Fe_3_O_4_ membranes with different nanoparticles’ concentrations. The figure reveals a decrease in resistivity (i.e., increase in conductivity) with increasing temperatures. At 25 °C, increasing the nanoparticles’ concentrations decreased the resistivity. In contrast, the resistivity increased the increasing nanoparticles’ concentrations at 75 and 100 °C. This observation was in agreement with the reported results of other researchers [37]. A better understanding of this dependence of the resistivity on nanoparticles’ contents can be extracted by the calculation of the activation energy ($E_{a}$). Therefore, the results were fitted into linear equations, as presented by the solid lines in the figure. The fitting lines revealed that, other than the 0% membrane, increasing the nanoparticles’ concentrations reduced the slope of the curve. The slope of a fitting line can be used to extract the activation energy by utilizing the Arrhenius equation: $\rho =\rho_{0}e^{\frac{E_{a}}{k_{B}T}}$, with $\rho_{0}$ a temperature-independent constant and $k_{B}$ representing the Boltzmann constant. The temperature (T) was measured in the unit of Kelvin. The dependence of the activation energy on the nanoparticles’ concentrations of PVA-GL-Fe_3_O_4_ membranes is presented in Figure 6b. Other than the 0% PVA-GL-Fe_3_O_4_ membrane, the figure revealed a decrease of the activation energy with the nanoparticles’ concentrations. This decrease in the activation energy referred to the rise in the concentration of the charge carriers as a result of the increasing nanoparticles’ concentrations. Increasing the nanoparticles’ concentrations facilitated the charge transport within the polymer nanoparticles matrix. Herein, the nanoparticles represented a network path for the charges transport to flow through. The resistivity of a pure PVA + GL membrane was balanced by adding Fe_3_O_4_ nanoparticles that were semiconductor in nature. Since the values of resistivity for the PVA + GL and nanoparticles were comparable, the addition of low concentrations of nanoparticles did not contribute to enhancing the electrical conductivity, but instead, they disturbed the charge conduction paths inside the membranes. The semiconducting influence of the nanoparticles became more dominant at high concentrations.

## 3. Experimental

### 3.1. Materials

PVA with 61,000 g/mol molecular weight, glycerol with 99.5% purity, ferrous chloride (FeCl_2_.4H_2_O_,_ 99%), ferric chloride (FeCl_3_.6H_2_O, 99%), and sodium hydroxide(98%) were purchased from Sigma Aldrich (82024 Taufkirchen, Germany), while tri-sodium-citrate was purchased from BDH (Kuwait City, Kuwait). The water used for the preparation of the PVA solution was double-distilled.

### 3.2. Synthesis of Nanoparticles

Fe_3_O_4_ nanoparticles were typically prepared by the simple coprecipitation method as described in reference [38] with some modifications. In particular, two equal volumes of 0.1 M FeCl_2_ and 0.2 M FeCl_3_ aqueous solutions with 1 wt% of trisodium citrate were poured into a glass beaker under mechanical stirring. During stirring, the pH of the mixture was adjusted to 9 by 2 M NaOH aqueous solution. The product, faint brown, was collected by centrifugation at 7000 rpm. The product was washed by ethanol and deionized water three times, then dried at 80 °C overnight.

### 3.3. Membrane Preparation

Composite flexible membranes were fabricated by utilizing a solution casting technique. Herein, a solution of 10 wt% of PVA was generated by dissolving granules of PVA (10 g) inside 100 mL of distilled water at a temperature of 80 °C under rigorous stirring. Once PVA granules were dissolved completely, ethanol (50 mL) was introduced to the solution while still stirring, where a solution of viscous nature was obtained. Glycerol with a concentration of 1 wt% was introduced to the PVA solution as a plasticizer. Nanoparticles were added to the PVA solution while the solution was placed on a solicitor for 30 min. This process is essential to guarantee the uniform dispersion of nanoparticles within the membranes. The PVA-GL-Fe_3_O_4_ solution was casted on top of aluminum foil and left to dry inside an oven at 80 °C for more than 4 h under ambient air.

## 4. Characterization

The sizes of the nanoparticles, morphology, and composition were tested using a Nova-NanoSEM-450 scanning electron microscope (SEM) (FEI, Lausanne, Switzerland) attached with an apparatus of energy-dispersive x-ray spectroscopy (EDS). Fourier-transform infrared (FTIR) spectrometer of PerkinElmer, spectrum-400-FT-IR/FT-NIR (Waltham, MA, USA), was utilized to examine the structure, as well as the vibration modes of the membranes. A Raman spectrometer of Thermo Fisher Scientific DXR (Waltham, MA, USA) was utilized to investigate the crystallinity of the membranes. PerkinElmer systems of differential scanning calorimeter (DSC) (model number Jade-DSC) and thermal gravimetric analysis (TGA) (model number Pyris6-TGA) were used to investigate the stability, melting point, and crystallinity indices of the membranes. The TGA was performed at a heating rate of 10 °C/min between 20 to 700 °C, while the DSC measurements were performed in a temperature range between 20 to 250 °C. A PANalytical x-ray diffraction (XRD) system of Empyrean was used to investigate the composition and structure of the produced nanoparticles by utilizing the Cu-$K_{\alpha }$ radiation peak of wavelength λ=1.5406 Å. The XRD measurements were applied in an angle (2θ) range of 10–80° with a 0.02° angle step size.

An impedance-gain-phase analysis system of Solarton (model number 1260A) was utilized to study the electrical characteristics of the membranes. The membranes were tested by adopting a capacitor scheme, where each membrane was located between a pair of electrical electrodes that were made of stainless steel on a test stage with temperature control. The electrical characterizations were established as a function of temperature with frequency range between 1–10^6^ Hz. The Solarton system detected electrical impedance as a function of frequency (f) of the ac signal (Z(ω)), where ω=2πf. The system also identified the phase angle that was a function of frequency (θ(ω)). The electrical impedance could be expressed in terms of real and imaginary parts $Z^{\prime }\left(\omega \right)$ and $Z^{\prime \prime }\left(\omega \right)$, respectively, where $Z\left(\omega \right)=Z^{\prime }\left(\omega \right)-iZ^{\prime \prime }\left(\omega \right),$ with i defined as the complex number. The impedance components were depicted on Nyquist plots with frequency as a tacit variable using Zview software. The Zview was also utilized to fit the impedance measurements and determine the equivalent resistance.

## 5. Conclusions

Flexible polymer membranes of poly(vinyl alcohol) (PVA), glycerol (GL), and iron oxide (Fe_3_O_4_) nanoparticles were fabricated and characterized. The nanoparticles were produced using a coprecipitation method with an average size of 20±9 nm, then added to PVA-GL to produce solutions with different nanoparticles’ concentrations. Flexible membranes were then fabricated by utilizing a solution casting method. X-ray diffraction measurements demonstrated the formation of Fe_3_O_4_ nanoparticles of cubic structures. The composition of nanoparticles was further assured using energy-dispersive x-ray spectroscopy (EDS) measurements. Differential scanning calorimetry (DSC), Fourier-transform infrared (FTIR) spectroscopy, and Raman analysis revealed that PVA membranes maintained their crystallinity with the increasing nanoparticles’ concentrations. The thermogravimetric calorimetry (TGA) demonstrated a decent stability of the prepared membranes. Electrical impedance characterizations demonstrated that the membranes may be presented as a single parallel RC circuit. The resistivity of the membranes exhibited a negative temperature coefficient, and it decreased with the nanoparticles’ concentrations. This caused a decrease of the activation energy with the nanoparticles’ concentrations because of the decrease in the scattering of charge carriers through the grain boundaries. The resistivity of the fabricated flexible membranes was viable to alter by introducing suitable concentrations of the nanoparticles. Hence, the generated membranes may be nominated as a potential flexible material for electronic flexible devices.

## Data Availability

Data is contained within the article. The data presented in this study are available in article.
